# Auraptene Boosts the Efficacy of the Tamoxifen Metabolites Endoxifen and 4-OH-Tamoxifen in a Chemoresistant ER+ Breast Cancer Model

**DOI:** 10.3390/pharmaceutics16091179

**Published:** 2024-09-06

**Authors:** Angel Pulido-Capiz, Brenda Chimal-Vega, Luis Pablo Avila-Barrientos, Alondra Campos-Valenzuela, Raúl Díaz-Molina, Raquel Muñiz-Salazar, Octavio Galindo-Hernández, Victor García-González

**Affiliations:** 1 Departamento de Bioquímica, Facultad de Medicina Mexicali, Universidad Autónoma de Baja California, Mexicali 21000, Mexico; pulido.angel@uabc.edu.mx (A.P.-C.); brenda.chimal@uabc.edu.mx (B.C.-V.); campos.alondra85@uabc.edu.mx (A.C.-V.); rauldiaz@uabc.edu.mx (R.D.-M.); octavio.galindo@uabc.edu.mx (O.G.-H.); 2 Laboratorio Multidisciplinario de Estudios Metabólicos y Cáncer, Universidad Autónoma de Baja California, Mexicali 21000, Mexico; 3 Max-Planck-Institute of Molecular Plant Physiology, Am Mühlenberg 1, 14476 Potsdam, Germany; luispablo.barrientos@mpimp-golm.mpg.de; 4 Escuela de Ciencias de la Salud, Universidad Autónoma de Baja California, Campus Ensenada, Ensenada 22890, Mexico; ramusal@uabc.edu.mx

**Keywords:** auraptene, breast cancer, estrogen receptor, resistance, eukaryotic initiation factor-4A complex

## Abstract

Approximately 80% of breast cancer (BC) cases are estrogen receptor positive (ER+) and sensitive to hormone treatment; Tamoxifen is a prodrug, and its main plasmatic active metabolites are 4-hydroxytamoxifen (4-OH Tam) and endoxifen. Despite the effectiveness of tamoxifen therapy, resistance can be developed. An increment in eukaryotic initiation factor-4A complex (eIF4A) activity can result in tamoxifen-resistant tumor cells. For this work, we developed a cell variant resistant to 4-OH Tam and endoxifen, denominated MCF-7^Var E^; then, the aim of this research was to reverse the acquired resistance of this variant to tamoxifen metabolites by incorporating the natural compound auraptene. Combination treatments of tamoxifen derivatives and auraptene successfully sensitized the chemoresistant MCF-7^Var E^. Our data suggest a dual regulation of eIF4A and ER by auraptene. Joint treatments of 4-OH Tam and endoxifen with auraptene identified a novel focus for chemoresistance disruption. Synergy was observed using the auraptene molecule and tamoxifen-derived metabolites, which induced a sensitization in MCF-7^Var E^ cells and ERα parental cells that was not observed in triple-negative breast cancer cells (TNBC). Our results suggest a synergistic effect between auraptene and tamoxifen metabolites in a resistant ER+ breast cancer model, which could represent the first step to achieving a pharmacologic strategy.

## 1. Introduction

Breast cancer (BC) is a heterogeneous disease classified into the following molecular subtypes: estrogen receptor positive α (ERα), epidermal growth factor receptor 2 positive (HER2+), and triple negative (TNBC) [1]. Approximately 80% of BC cases are estrogen receptor positive (ER+) [1]. One of the most widely used drugs for the treatment of BC ER+ is tamoxifen, a selective ER modulator that competes with its natural ligand, estradiol (E2), an estrogen hormone playing a critical role in breast tissue growth, development, and function [2]. Tamoxifen is classified as a prodrug due to its transformation into active metabolites upon metabolism. These metabolites include N-desmethyltamoxifen (NMD), 4-hydroxy-N-desmethyltamoxifen (endoxifen), and 4-hydroxytamoxifen (4-OH Tam), which exhibit significantly 30 to 100 times greater affinity for ER compared to tamoxifen itself [3].

Although one of the most recurrent treatments against ER+ BC is tamoxifen, some patients develop a resistance to this drug. Particularly, there are reports on the acquisition of 4-OH Tam resistance for ER inhibition [3]; however, there is limited information available regarding resistance to the more potent metabolite, endoxifen. Moreover, 4-OH Tam and endoxifen are the main plasmatic metabolites. The emergence of chemoresistance at various stages has significantly increased the complexity of BC treatment [4].

Several mechanisms have been elucidated to explain how neoplastic cells develop resistance to therapy. These include diminished prodrug activation, heightened expression of efflux pumps, reduced drug internalization [5], and the activation of enzymatic systems responsible for drug catabolism.

Likewise, one of the causes of resistance to tamoxifen is the decrease in ER expression through transcriptional suppression, causing a phenotype transformation or changes in the percentage of cell populations among low and normal expression of ER [6]. A low concentration of intratumoral tamoxifen metabolites has also been observed in some cases of tamoxifen-resistant BC, suggesting a condition of acquired resistance associated with an increase in ATP-binding cassette (ABC) or Multidrug resistance (MDR) efflux pumps [7]. ERα is a key player in the context of ER+ breast cancer, influencing both pathogenesis and treatment response. Moreover, ERα signaling exhibits intricate cross-talk with other pathways, such as those mediated by growth factor receptors, influencing cell growth and survival [8]. 

This interaction leads to an increase in selective protein synthesis, making the eukaryotic initiation factor 4F complex (eIF4F) an important target [9,10]. The eIF4F complex is composed of three key components, eIF4E, eIF4A, and eIF4G, which together coordinate the initiation of mRNA translation in eukaryotic cells. An overexpression or hyperactivation of factor eIF4E has been implicated in the translation of mRNA targets including cyclin D1, c-Myc, VEGF, and Bcl-2, which are known to depend on ER signaling and contribute to BC progression, and also the increase in translation of pro-survival proteins, including those involved in drug resistance mechanisms such as anti-apoptosis and drug efflux pumps. Moreover, aberrant eIF4G signaling can enhance the assembly and stability of the eIF4F complex, leading to the increased translation of mRNA. In addition, alterations that increase the expression or enhance the helicase function of eIF4A can lead to the translation of specific mRNA transcripts, thereby potentiating chemoresistance according to our results [11,12]. Together, these components form a dynamic complex crucial for efficient mRNA translation, whose dysregulation is often implicated in chemoresistance phenomena registered in cancer cells [13]. Through its influence on the translation of these key proteins, the eIF4F complex can impact various aspects of ER-mediated cellular processes, including cell cycle regulation, proliferation, angiogenesis, and apoptosis resistance. Moreover, in MCF-7 cells (ER+ cells), heightened expression levels of ABCB1 and ABCC1 efflux pumps have been described [14]. Therefore, this dual regulation eIF4F and ER could represent a strategic therapeutic approach for ER chemoresistance.

Natural compounds have potential as a therapeutic strategy. In this regard, several molecules such as rocaglates, hippuristanol, and pateamine A have been reported to have anticancer activity in vitro and in vivo models [15]. Indeed, rocaglamide and silvestrol have been characterized as adjuvant compounds in cancer treatment, capable of inhibiting the subunit eIF4A [16,17,18]. However, new molecules of natural origin may represent a potential opportunity area; in this regard, auraptene, a prenyloxycoumarin obtained from plants of the genus *Citrus*, could show several pharmacological properties, such as being an antineoplastic agent, apoptosis inducer, neuroprotective agent, metalloproteinase inhibitor, and an antioxidant and hepatoprotective agent. Auraptene has also been proposed to bind to ERα and modulate its transcriptional activity in ER+ cells [19]. Therefore, auraptene is proposed as a molecule with a dual function in the modulation of strategic protein targets in ER cancer cells, moreover, in chemoresistance conditions.

Natural compounds, the bioactive molecules derived from plants, herbs, and other sources, have demonstrated a therapeutic anti-cancer effect, ranging from anti-inflammatory and antioxidant properties to immune modulation and apoptosis induction. Tamoxifen metabolites used along these natural compounds may have a potential to counteract chemoresistance mechanisms and enhance therapeutic outcomes. In this regard, we explored the use of auraptene for developing improved treatment strategies targeting ER signaling in breast cancer, as well as its use in chemoresistance conditions.

## 2. Materials and Methods

### 2.1. Materials

The salts and buffers were obtained from Merck (Darmstadt, Germany). The cell culture reagents were purchased from Thermo-Fisher (Carlsbad, CA, USA); tissue culture plates and other plastic materials were obtained from Corning Inc. (Corning, NY, USA). For the MTT reactive, endoxifen, 4-OH Tam, and aurpatene (Aur) were obtained from Merck in a concentration of ≥98% (HPLC). Anti-β actin (sc-8432), anti-eIF4A (sc-377315), anti-eIF4E (sc-271480), anti-eIF4G (sc-133155), anti-RE (sc-8002), anti-Cathepsin D (sc-377299), anti-GADPH (sc-32233), and anti-p-4E-BP1/2/3 (sc-271947) antibodies were obtained from Santa Cruz Biotechnology (Dallas, TX, USA). The horseradish peroxidase-conjugated anti-mouse secondary antibody from Thermo-Fisher was used for detection using the immobilon western kit (Millipore Western from Millipore, Burlington, MA, USA) [20]. β-actin and GAPDH were used as loading controls. 

### 2.2. Cell Culture

MCF-7 cells, ERα and PR positive, were purchased from American Type Culture Collection (ATCC, Manassas, VA, USA), accession number: HTB-22. The TNBC cell line model MDA-MB-231 was purchased from American Type Culture Collection (ATCC, Manassas, VA, USA), accession number: HTB-26. Cell cultures were grown in DMEM medium supplemented with 10% fetal bovine serum (FBS), 10 U/mL penicillin, 10 µg/mL streptomycin, and 25 µg/mL amphotericin B; 1100 units of insulin were added for MCF-7 according to ATCC recommendations. Cultures were maintained at 37 °C in a humidified atmosphere with 95% air and 5% CO_2_. The culture medium was changed every 3 to 4 days according to ATCC recommendations.

### 2.3. Chemoresistance Protocol

To generate a drug-resistant phenotype in MCF-7 cells, we established a protocol for resistance development to the active metabolites 4-OH Tam and endoxifen. Cells were treated with 1 µM concentrations of each metabolite and supplemented with 2 µM estradiol. Stock solutions of the metabolites were prepared at 1 mM in DMSO for endoxifen and estradiol, while 4-OH Tam was prepared in ethanol.

The treatment protocol involved exposing the cells to 1 µM of 4-OH Tam and endoxifen for 48 h; these concentrations of the metabolites were also used by Calley et al. [21], but they were applied independently. Following this treatment, a recovery period was implemented wherein the cells were maintained in fresh medium free of 4-OH Tam and endoxifen for 24 h. Subsequently, the cells were again treated with 1 µM of each metabolite plus 2 µM of estradiol for another 48 h, maintaining a molar 1:1 ratio of each metabolite. This cycle was repeated for a duration of 4 months to induce resistance. For maintaining the resistant cell variant, the culture was maintained with 50 nM of 4-OH Tam and endoxifen. This maintenance protocol was adapted from Chen et al. (2020) [22].

### 2.4. Cell Viability

Cell viability was assessed using the MTT assay according to a previous protocol [23]. The experiments were performed in a 96-well plate, seeding 20,000 cells, which were incubated to reach 80% of confluence. Next, cells were incubated under different treatments. Formazan crystals were dissolved in a lysis buffer containing 20% SDS and 50% N,N-dimethylformamide (pH 3.7) for 12 h at 37 °C. Optical densities were measured at 570 nm using a microplate reader.

The treatments used in the cell viability assays were under 24 h incubation periods using the following increasing concentrations: 0–16 μM for endoxifen, 4-OH Tam, and fulvestrant. For auraptene, the concentrations used were 0–100 μM, and for joint treatments, endoxifen and 4-OH Tam was used in a concentration of 8 μM with 12 h of incubation.

To determine EC50 values, the software GraphPad Prism 8 was used. The obtained data were transformed and normalized, followed by the application of a five-parameter asymmetric curve model.

### 2.5. Western Blot (WB) Analysis

Cells were seeded at a density of 200,000 cells/mL in 20 mm 6-well plates and incubated until they reached 90% confluence. After reaching the desired confluence, cells were exposed to the specified treatments. Subsequently, cells were washed with PBS and lysed for 35 min at 4 °C using a protein lysis buffer containing protease and phosphatase inhibitors. The lysates were centrifuged at 4100× *g* for 10 min, and the supernatant was collected. Protein quantification was performed using a BCA assay.

Samples (12 μg/lane) from the total protein fraction were analyzed using SDS-polyacrylamide gel electrophoresis (SDS-PAGE) using 8–12% gels, depending on the molecular weight of the target proteins. Proteins were transferred to PVDF membranes (Millipore, Burlington, MA, USA). Membranes were blocked with 5% nonfat milk in Tris-buffered saline with 0.1% Tween-20 (TBS-T) for 1 h at 37 °C, followed by overnight incubation at 4 °C with the respective primary antibodies: anti-ER (1:250), anti-β actin (1:500), anti-eIF4A (1:500), anti-eIF4E (1:400), anti-GAPDH (1:500), anti-Cathepsin D (1:450), anti-eIF4G (1:300), and anti-p-4E-BP1/2/3 (1:400).

After washing with TBS-T, membranes were incubated for 2 h at 37 °C with the corresponding horseradish peroxidase (HRP)-conjugated secondary antibodies. Membranes were washed again with TBS-T, and HRP activity was detected using the Immobilon Western kit (Millipore, MA, USA). Immunoblots were analyzed using the ImageJ 1.51 program, and the figures presented are representative of the blots.

### 2.6. Optical Microscopy

Cells were seeded at a density of 200,000 cells/mL in 6-well plates of 20 mm and were subsequently proliferated until 90% of confluence. Next, cells were incubated under indicated treatments. We utilized an inverted microscope VWR Vista Vision coupled to a camera Moticam 5 (Vancouver, BC, Canada), and cell imaging data were analyzed using Motic Images plus 3.0.

### 2.7. Molecular Docking

The three-dimensional structure of eIF4A1 was obtained from the Protein Data Bank (PDB) [24] ID 5ZC9, which corresponds to the structure of human eIF4A1-ATP at 2 Å resolution. The PDB 3ERT three-dimensional structure of ERα at 1.9 Å resolution was used. The structures of the ligand molecules were obtained from the PubChem database [25], rocaglamide (CID 331783), auraptene (CID 1550607), endoxifen (CID 10090750), 4-OH Tam (CID 449459), and fulvestrant (CID 104741). The protein structures were prepared by removing water and small molecules, leaving only the protein structure. The ligand and receptor were protonated in 3D and energy minimization; these experiments were performed using Molecular Operating Environment (MOE) 2022.02 software [26] with default parameters under the AMBER99 force field [27]. For the ligands, different conformations were generated using a stochastic search on the MOE default parameters.

Binding sites were predicted by employing the site finder option of the MOE software [28]. Molecular docking was established with the default parameters of MOE software, and refinement was used. For interpretation of the docking results, MOE identifies salt bridges, hydrogen bonds, hydrophobic interactions, sulfur-LP, cation-π, and solvent exposure, and gives the score S as a value of affinity ligand–receptor. Ligand interactions with target proteins were predicted based on the S score [29].

To find inaccessible binding modes with MOE’s scoring function, molecular docking simulations were also carried out using Vina [30]. Protein and ligand structures were prepared using Chimera’s [31] Dockprep plugin. Polar hydrogens were added, Gasteiger charges were calculated with ANTECHAMBER [32] and AMBER ff99sb force field, and missing residues were added using Dunbrack’s rotamer library [33]. Structures were then minimized using 100 steps of steepest descent with step sizes of 0.02 Å followed by 10 conjugate gradient steps of the same step size. Autodock Vina was then used to prepare pdbqt files and run the docking simulations using a grid box of enough volume to contain both the protein and the ligand and with an exhaustiveness parameter of 8.

### 2.8. Overexpression and Purification of eIF4A1

The gene coding for the eIF4A1 protein was cloned into a modified pET19b expression vector (pET19bm), which contains an ampicillin resistance gene, a polylinker site, the lac operon sequence controlled by the T7 promoter, and a 10-histidine tract at the N-terminal of eIF4A1. Additionally, the vector includes a proteolysis site for Prescission Protease (PPS, GE Healthcare, Chicago, IL, USA) to facilitate the removal of the histidine tract.

The plasmid was transformed into *Escherichia coli* Rosetta Star cells (Novagen, Darmstadt, Germany) harboring the pET19b-eIF4A1 plasmid. Cultures were grown at 37 °C in 2XYT medium supplemented with ampicillin (100 µg/mL) until an optical density (OD600) of 0.6 was reached. Protein expression was induced by adding Isopropil β-D-1-thiogalactopyranoside (IPTG) to a final concentration of 1 mM, followed by incubation for 16–24 h at 37 °C.

After incubation, the cells were collected using centrifugation and lysed using sonication. The supernatant was obtained and washed with a buffer containing 50 mM NaH_2_PO_4_, 300 mM NaCl, and 10 mM imidazole. Purification of the eIF4A1 protein was carried out using immobilized metal affinity chromatography (IMAC) with Ni-NTA agarose resin (QIAGEN, Hilden, Germany). Purification was evaluated using native acrylamide gel adapted from Arndt Cluadia et al. protocol [34], and the identification was performed using Western blot.

### 2.9. Fluorescence Assays for Auraptene-eIF4A Binding

Measurements were performed using a Cary Eclipse fluorometer (Mulgrave, VIC, Australia) scanning from 250 to 350 nm at 25 °C in a synchronous mode. Protein–ligand interactions were evaluated using 12 µM eIF4A1 and a range of auraptene concentration 0–80 μM. Solutions were homogenized and incubated for 5 min at 25 °C, and measurements were performed in a quartz cell with a path length of 1 cm and 500 µL volume at 25 °C.

### 2.10. ATPase Activity Assay for eIF4AI

The ATPase assay in polyacrylamide gel with lead nitrate (PbNO_3_) staining was employed to evaluate the effect of auraptene on eIF4A ATPase activity. Serial dilutions of auraptene were prepared through a concentration range of 0–200 μM. Rocaglamide, used as a control, was prepared at a concentration of 5 nM based on a previous report [17]. After performing gel electrophoresis, the gels were stained with lead nitrate. Then, ATPase activity was visualized as clear bands against a dark background.

### 2.11. qPCR for ABCC1 and ABCB1 Expression

Cells were seeded at a density of 200,000 cells/mL in 6-well plates of 20 mm and were subsequently incubated until 90% confluence was reached. Next, cells were incubated under indicated treatments for 12 h. The total RNA from cell variants were obtained with Trizol reagent, following the supplier’s instructions. cDNA was synthesized using 1 µg of RNA and the Primer Script RT-PCR. cDNA concentration was standardized for qPCR with the PowerUp Sybr Green Master Mix 2X (Applied Biosystems, Waltham, MA, USA) according to the manufacturer’s instructions, and 10 μL of the resultant cDNAs was used for each PCR reaction. Primer sequences were *ABCB1* forward 5′-GCCAGCTGAACTCCTTAGAC-3′; *ABCB1* reverse 5′-GATTCGTGCACAGCAGCA-3′; *ABCC1* forward 5′-GGCTCAAGGAGTATTCAGAG-3′; *ABCC1* reverse 5′-CCATCGATGATGATCTCTCC-3′; *GAPDH* forward 5′-AGACAGCCGCATCTTCTTGT-3′; and *GADPH* reverse 5′-CTTGCCGTGGGTAGAGTCAT-3′. qPCR reactions were performed in QuantStudio 1 by applied biosystems. Data were analyzed with the 2^−∆∆Ct^ method with GAPDH as reference, and results were reported as fold change. 

### 2.12. ADME Properties of Auraptene

The chemical structure of auraptene was input into the SwissADME [35] web tool and the ADMETlab 2.0 [36] interface to identify favorable ADME characteristics. The ADME (Absorption, Distribution, Metabolism, and Excretion) properties of auraptene determine its drug-likeness and desired pharmacokinetic profiles. Specifically, we focused on evaluating optimal lipophilicity, high water solubility, and predicted bioavailability.

### 2.13. Statistical Analysis

All data are expressed as mean ± standard deviation (SD). Statistical analyses were performed using two-way analysis of variance (ANOVA) with GraphPad Prism 8. For MTT assays, the data are also expressed as mean ± SD. According to GraphPad Prism, manual results were considered statistically significant if *p* < 0.05.

## 3. Results

### 3.1. Resistance Acquisition under Tamoxifen-Derived Metabolites and Estradiol Treatments in ERα Cells

**Figure 1:**
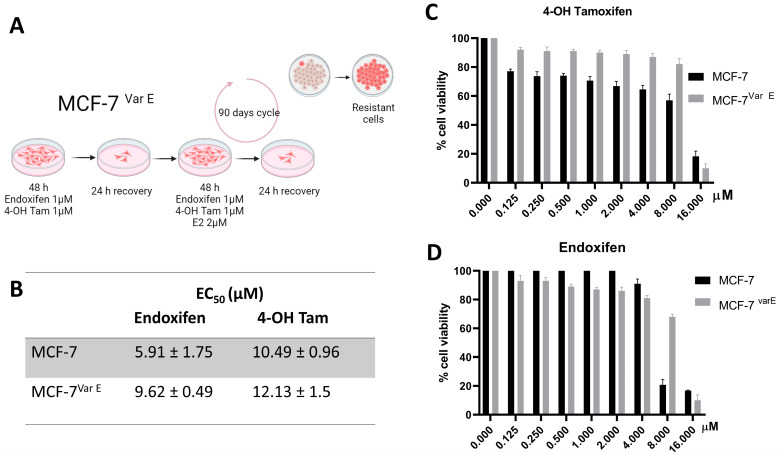
Resistance development in MCF-7 cells and its characterization. (**A**) Illustration of the protocol used to carry out chemoresistance, showing the procedure for resistance acquisition in MCF-7^Var E^ cells. (**B**) Comparison of the EC_50_ values and SD among MCF-7 and MCF-7^var E^ cells under the 4-OH Tam (**C**) and endoxifen (**D**) treatments (0–16 µM).

An MCF-7 (ER+) cell variant resistant to 4-OH Tamoxifen (4-OH Tam) and endoxifen was generated, denominated as MCF-7^Var E^. The endocrine resistance was acquired through treatment under a scheme of 1 μM of endoxifen, 1 μM 4-OH Tam, and 2 μM estradiol (E2) in a concomitant incubation; E2 was utilized as an antagonist against the tamoxifen metabolites. The ratio of active metabolites to hormones was maintained at an equimolar concentration. The resistant variant was generated by stimulating the cell cultures with Tamoxifen metabolites for 48 h periods followed by a 24 h recovery period; maintenance doses of 50 nM of each compound were incubated over a 120-day cycle to induce endocrine resistance (Figure 1A). Cell viability experiments were performed to determine the endocrine resistance of the MCF-7^Var E^ variant. Using the MTT assay, the half maximal effective concentration (EC_50_) of each active metabolite for MCF-7 and MCF-7^var E^ cell cultures was quantified to determine the resistance of the variants (Figure 1B–D).

The MCF-7^Var E^ showed an increase in EC_50_ values under treatment with endoxifen (9.62 ± 0.49 µM) and 4-OH Tam (12.13 ± 1.5 µM) in comparison to MCF-7 cells with values for endoxifen (5.91 ± 1.75 µM) and 4-OH Tam (10.49 ± 0.96 µM) (Figure 1B–D). Results demonstrate the acquisition of resistance to the pharmacologically active tamoxifen metabolites. In a complementary way, the experimentation with the fulvestrant drug showed a similar behavior; an increase in the EC_50_ value for the MCF-7^Var E^ (29.62 ± 0.49 µM) compared to MCF-7 cells (19.81 ± 0.49) was registered (Supplementary Figure S1).

### 3.2. Effect of Estradiol as a Determinant of Chemoresistance

E2 functions as a critical regulator of ERα signaling. Although its presence promotes cell growth and proliferation, elucidating the effects of E2 is crucial for unraveling the complexities of ER+ breast cancer regulation and for developing therapeutic strategies.

**Figure 2:**
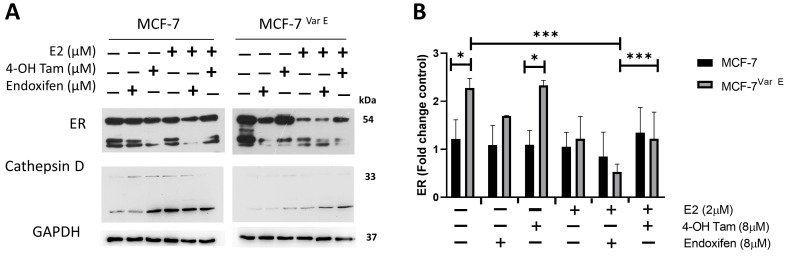
Role of estrogen receptor (ER) in chemoresistance. (**A**) Expression of ER and cathepsin D. The comparative expression between MCF-7 and MCF-7^Var E^ under the indicating conditions, with E2 (2 µM) and tamoxifen metabolites treatment (8 µM). (**B**) Quantitative analysis of ER expression. Comparative expression by densitometry analysis in three independent experiments of ER in MCF-7 and MCF-7^Var E^ cells. Results are reported as mean ± SD (*n* = 3) and expressed as fold-change in regard to loading control; * *p* < 0.05, *** *p* < 0.001 in regard to control. GAPDH were used as loading controls.

Under conditions of chemoresistance, tamoxifen metabolites induced an increase in ER expression in the MCF-7^Var E^ in comparison to MCF-7 cells under basal conditions (Figure 2A,B). Moreover, combined treatment with E2- and tamoxifen-derived metabolites showed a down-regulation in ERα expression (Figure 2A,B), which in turn caused more significant cell damage (Supplementary Figure S2). This phenomenon could be associated with the fact that E2 stimulation increases cellular activity, enhancing the effectiveness of tamoxifen metabolites, particularly under the combined treatments. In addition, in the resistant MCF-7^Var E^ cells, the combined effect was more pronounced, resulting in a significant decrease in ERα expression (Figure 2A,B) and, consequently, a more potent cytotoxic effect (Supplementary Figure S2). These results suggest a role of ERα under elevated concentrations of pharmacologically active tamoxifen-derived metabolites.

Given the impact of changes in cathepsin D expression levels on breast cancer prognosis [37], we also determined its expression in control and resistant cells. Importantly, we observed an increase in cathepsin D levels in both MCF-7 and MCF-7^Var E^ cells when treated with a combination of estradiol and tamoxifen metabolites.

Additionally, we observed an association between the resistant phenotype and increased ER expression. To investigate whether tamoxifen metabolites could modulate ER activity, we conducted molecular docking simulations (Supplementary Figure S3A–C). The obtained affinity values through algorithm MOE (Molecular Operating Environment), E-scores, were −5.60 kcal/mol for estradiol, −7.81 for endoxifen, and −7.29 for 4-OH Tam, suggesting a higher affinity for tamoxifen-derived metabolites on ER structure. 

Notably, these results revealed that the binding site of tamoxifen-derived metabolites overlaps with the interaction region of E2, suggesting a potential competition phenomenon for the ER-native ligand-binding domain (LBD); indeed, several residues involved in E2 binding, such as Glu_380_, Leu_536_, and Leu_525_, are also involved in binding both endoxifen and 4-OH Tam (Supplementary Figure S3D–G).

Ligand/tamoxifen-derived molecules competing for ER could favor a potent inhibitory effect, inhibiting cell proliferation by down-regulating the translation of ER downstream genes. Under these conditions, adaptive mechanisms must be triggered to maintain chemoresistance; in this regard, targeting the eIF4F complex and its associated signaling pathways have emerged as potential therapeutic strategies to overcome ER+ cancer chemoresistance [38]. 

### 3.3. eIF4F Protein Components Are Conserved during Chemoresistance and through Treatment of Tamoxifen-Derived Metabolites Plus E2

**Figure 3:**
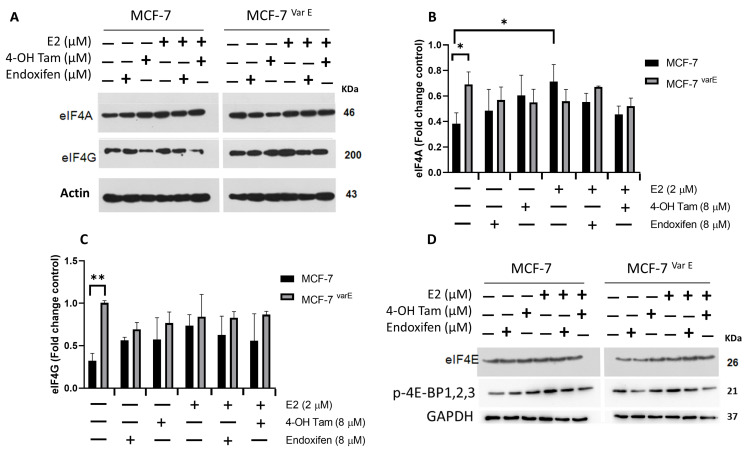
eIF4F complex characterization. (**A**) Expression of eIF4A and eIF4G targets. The comparative expression between MCF-7 and MCF-7^Var E^ cells under different treatments, with E2 (2 µM) and tamoxifen metabolites (8 µM) for 12 h. Densitometry analysis of eIF4A (**B**) and eIF4G (**C**) in MCF-7 and MCF-7^Var E^ cells under the same conditions. (**D**) Expression of eIF4E and p-4E-BP1,2,3. The comparative expression between MCF-7 and MCF-7^Var E^ under different treatments with E2 and tamoxifen metabolites is shown. Results are reported as mean ± SD (*n* = 3) and expressed as fold-change in regard to loading control; * *p* < 0.05, ** *p* < 0.002 in regard to control. β-actin and GAPDH were used as loading controls.

To determine the role of the eIF4F complex in ER drug resistance cells, we characterized its expression in MCF-7 and MCF-7^Var E^ cells. In the first instance, for eIF4A, the RNA-helicase protein MCF-7^Var E^’s cells showed increased expression levels when compared to MCF-7 parental cells, possibly due to drug resistance (Figure 3A,B). Notwithstanding, eIF4A levels were higher in E2-treated MCF-7 cells compared to their control; in contrast, MCF-7^Var E^ cells show an eIF4A reduction with E2 treatment, suggesting that E2 has a differential effect on the cell variants. Interestingly, the levels of eIF4A were maintained despite the concomitant treatment of tamoxifen-derived metabolites and E2 (Figure 3A,B).

In the case of the eIF4G translation initiation factor, we obtained a similar result, an increased expression in MCF-7^Var E^ compared to the MCF-7 cells (Figure 3A,C); however, endoxifen and 4-OH Tam treatment (8 μM) induced a slight reduction in its expression in MCF-7^Var E^. Moreover, in MCF-7 cells, the same tamoxifen-derived treatments induced a slight increment (Figure 3A,C). In this case, the response was regulated by the E2 treatment, and the MCF-7^Var E^ cells had a more active complex that could confer chemoresistance (Figure 3A,C).

Regulation of eIF4E expression is crucial, with 4E-BP proteins modulating its activity by binding to eIF4E and inhibiting eIF4F complex formation. Interestingly, in MCF-7 cells, eIF4E expression remained unchanged despite tamoxifen metabolite stimuli (Figure 3D). Moreover, in MCF-7^Var E^ cells, eIF4E expression increased with concomitant treatments (Figure 3D). The phosphorylation status of p-4E-BP1,2,3 implies a potential eIF4E incorporation into the eIF4F complex, likely rendering it more active in MCF-7^Var E^ cells (Figure 3D). 

Evidence suggests a compensatory mechanism to maintain the activity of the complex by potentially increasing the expression of eIF4G and eIF4A. Moreover, the expression of the translation factor is maintained despite tamoxifen-derived treatment and concomitant treatment with E2. eIF4A could remain bound to the eIF4F complex and fulfill its function, a particular feature of the MCF-7^Var E^-resistant cells. This prompted us to search for strategies for eIF4F regulation, specifically eIF4A and Erα, through inhibition by natural compounds, such as coumarins.

### 3.4. Regulation of eIF4A by Auraptene Binding

**Figure 4:**
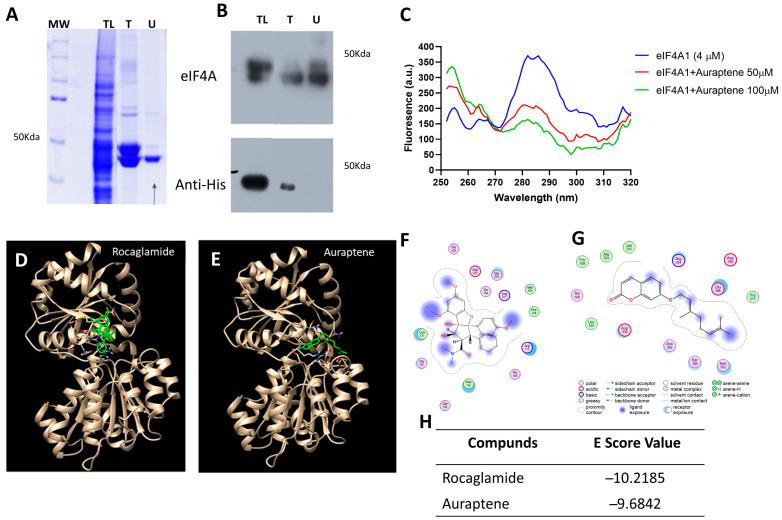
Effect of auraptene on the eIF4A regulation. (**A**) Coomassie-stained SDS-PAGE of eIF4A-purified fractions corresponding to TL: total lysate, T: tagged protein, and U: untagged protein. (**B**) Immunodetection for the fractions of eIF4A overexpression. Antibodies to detect eIF4A and polyhistidine tags were used. (**C**) Fluorescence assay of eIF4A and auraptene. Emission fluorescence spectra of eIF4A, eIF4A plus 50 µM auraptene, and eIF4A plus 100 µM auraptene are shown in blue, red, and green, respectively. (**D**) Docking of rocaglamide in the eIF4A RNA binding site (PDB ID: 5ZC9). eIF4A protein is shown in beige and rocaglamide molecule in green. (**E**) Docking of auraptene in the eIF4A RNA interaction site. eIF4A is shown in beige and auraptene in green. (**F**) Rocaglamide–eIF4A ligand interactions, showing the residues and type of interaction. (**G**) Auraptene–eIF4A binding residues and the nature of the interactions with molecules are shown. (**H**) E-score values obtained for each molecule in the molecular docking simulation.

Auraptene, a natural coumarin found in citrus fruits, could show promising potential in cancer treatment. According to our results, auraptene may disrupt the translation process through eIF4A inhibition, in turn affecting the expression of proteins involved in cell growth, survival, and potentially chemoresistance. To find if auraptene binds eIF4A, we overexpressed and purified recombinant eIF4A from *Escherichia coli* Rosetta Star strain cultures (Figure 4A,B). Results showed a significant decrease in fluorescence under the interaction of eIF4A (4 µM) and auraptene at concentrations of 50 μM and 100 μM; a reduction in the emission intensity was observed, resulting from a quenching phenomenon, suggesting that auraptene binds with the eIF4A protein (Figure 4C). Molecular docking simulation results suggest that Aur binds eIF4A close to the rocaglamide binding site, as different docking protocols found highly scored auraptene binding poses near the RNA binding site. Critical residues for rocaglamide interaction are also shared, such as Asp330, Leu331, Pro108, and Thr109 (Figure 4D–G). The E-score for values for auraptene was −9.68 Kcal/mol, which was close to rocaglamide’s −10.21 (Kcal/mol) (Figure 4H). Additionally, Vina’s scoring function allowed us to find an auraptene pose in which the lactone oxygen hydrogen bonds to Arg^334^ (Supplementary Figure S4).

### 3.5. Enhancing Chemotherapeutic Sensitivity in ER+ Cells with Auraptene Treatment

**Figure 5:**
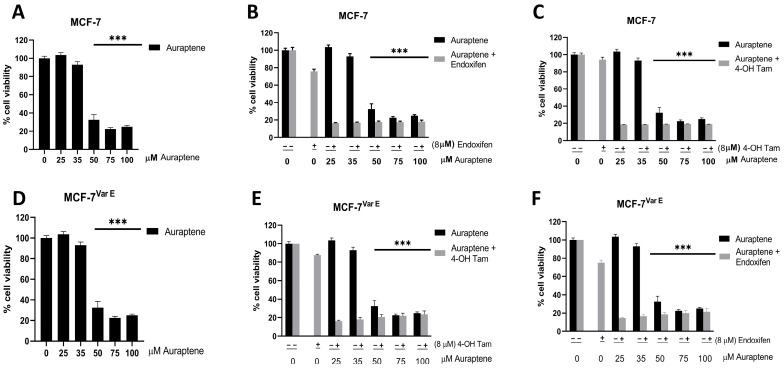
Cell viability of MCF-7 and MCF-7^Var E^ cells with auraptene treatments. Comparison of auraptene and auraptene plus tamoxifen metabolite treatments. Viability percentages of MCF-7 cells under auraptene (**A**), auraptene and 4-OH tamoxifen (**B**), and auraptene and endoxifen treatments (**C**). Results of auraptene and auraptene plus tamoxifen metabolites shown in black and gray columns, respectively. Viability percentages of MCF-7^Var E^ cells under auraptene (**D**), auraptene and 4-OH tamoxifen (**E**), and auraptene and endoxifen (**F**) treatments. Results of auraptene and auraptene plus tamoxifen metabolites shown in black and gray columns, respectively. Results are reported as mean ± SD (*n* = 3); *** *p* < 0.0001 regard to control.

Auraptene is a natural compound which has been proposed to have anticarcinogenic effects in several cancer cell lines [39]; however, it has not been studied in chemoresistant conditions. We thus set out to test the effects of auraptene on MCF-7^Var E^ and MCF-7 cells to evaluate potential synergistic effects with the active tamoxifen metabolites. We employed concentrations below their EC_50_ values (Figure 5A,D), and active metabolites of tamoxifen, endoxifen, and 4-OH Tam at a concentration of 8 µM (Figure 5). 

Our results revealed that auraptene significantly reduced cell viability starting from a 50 µM concentration in both MCF-7 and MCF-7^Var E^ (Figure 5A–D). Subsequently, we explored the combined treatment of auraptene with tamoxifen metabolites. Interestingly, auraptene exhibited a synergistic effect with both 4-OH Tam (Figure 5B,E) and endoxifen (Figure 5C,F), resulting in a substantial reduction in cell viability in both MCF-7 and MCF-7^Var E^ cells. Remarkably, this effect was observed even at the lowest auraptene concentration evaluated (25 µM), highlighting the potency of the auraptene–tamoxifen metabolite combination treatment.

Despite its chemoresistance acquisition, we observed a potential therapeutic effect in MCF-7^Var E^ cells. By modulating key signaling pathways involved in drug resistance mechanisms, auraptene exhibited the potential to enhance the efficacy of conventional treatments in ER+ breast cancer cells. Notably, the joint treatments were shown to be effective for both MCF-7 cells and the resistant variant MCF-7^Var E^. Nevertheless, this treatment may be specific to ER+ breast cancer. Thus, we evaluated this therapeutic effect in another breast cancer molecular subtype.

### 3.6. Auraptene Specificity in ER+ Breast Cancer Cells

**Figure 6:**
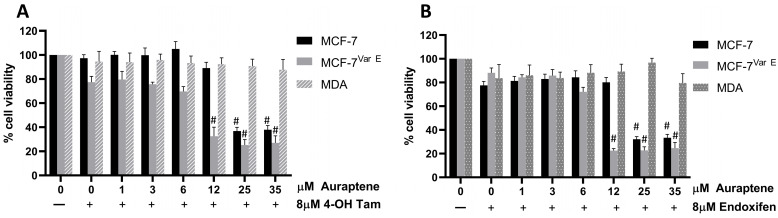
Auraptene and mixed auraptene plus tamoxifen metabolites treatments on ER+ and ER- cell variants. Comparative cell viability percentages of MCF-7, MCF-7^Var E^, and MDA-MB-231 cells are shown in black, light gray, and pattern gray, respectively, under increasing doses of auraptene plus 4-OH Tam 8 µM (**A**) and endoxifen 8 µM (**B**). Results are reported as mean ± SD (*n* = 3); # *p* < 0.0001 regard to control.

To elucidate if the effects of the combined treatments of auraptene with tamoxifen-derived metabolites are specific to BC cells that overexpress ER+, the triple-negative breast cancer cell model lacking expression of hormone receptors, MDA-MB-231 (MDA) cell line, which shows the main phenotypic properties of TNBC cancer, was evaluated. The MDA cell line displayed a higher tolerance to auraptene than the MCF-7 and MCF-7^Var E^ ER+ cell lines, with a viability of 80% observed at the auraptene concentration of 35 μM (Figure 6).

To determine the minimal auraptene concentration at which synergy with auraptene and tamoxifen metabolites could be registered, we used low concentrations of auraptene (0–35 µM) and a fixed concentration of tamoxifen metabolites (8 µM) (Figure 6A,B). Results suggest a sensitizing effect observed on both ER+ cell variants MCF-7 and MCF-7^Var E^ under the treatment with 4-OH Tam (Figure 6A, Table 1). In contrast, triple-negative cells showed higher tolerance to joint treatments (Figure 6A, Table 1). Both MCF-7 variants displayed synergy between auraptene and both tamoxifen metabolites, as evidenced by lower EC_50_ values for auraptene when cells were treated with tamoxifen metabolites. This synergy resulted in EC_50_ values almost four times lower for MCF-7 cells treated with auraptene and tamoxifen metabolites when compared to cells treated with auraptene (Figure 6A,B, Table 1); synergy was more pronounced in MCF-7^Var E^ cells, as concomitant treatments of tamoxifen metabolites and auraptene resulted in EC_50_ lowering by almost an order of magnitude when compared with cells treated with auraptene (Figure 6A,B, Table 1). However, MDA cells (TNBC) displayed no synergy between auraptene and tamoxifen metabolites, the EC_50_ value for auraptene was 76.75 µM and the concomitant treatment with 4-OH Tam did not induce a significant therapeutic effect, the EC_50_ value remained at 80.65 µM (Figure 6A, Table 1).

Results for endoxifen showed a synergistic effect on MCF-7 and MCF-7^Var E^ cells, but for the triple-negative cells, it did not show any significant effect (Figure 6B). We registered a reduction in EC_50_ values in both variants, while the EC_50_ for auraptene treatment in MCF-7 cells was 47.49 µM, the impact of endoxifen reduced the EC_50_ to 13.34 µM; for MCF-7^Var E^ cells, the values were 52.4 µM for auraptene treatment, while the concomitant use of endoxifen promoted a reduction to 6.47 µM in EC_50_. For MDA cells, the EC_50_ value for auraptene was 76.75 µM and the concomitant treatment with endoxifen did not induce a significant therapeutic effect; the EC_50_ value was maintained at 52.68 µM (Figure 6B, Table 1).

Our results suggest a therapeutic potential for the concomitant treatment for ER+ cells, which requires further testing. Auraptene displayed efficacy at concentrations as low as 12 μM, almost five times lower than the effective concentration of auraptene alone (50 μM) at 24 h of treatment. As a result, subsequent experiments were carried out at an auraptene concentration of 12.5 μM to elucidate the specific mechanism of sensitization on ER+ cell lines.

### 3.7. Characterization of Auraptene Molecular Mechanism of Resistance

**Figure 7:**
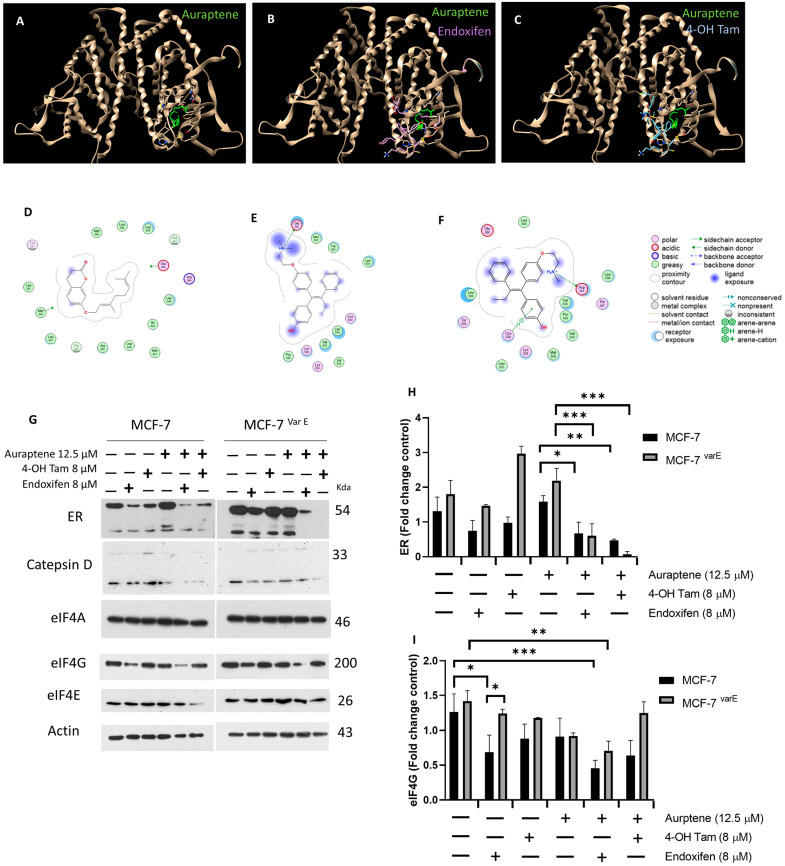
Characterization of eIF4F complex and ER. Molecular docking of molecules of auraptene and tamoxifen metabolites on estrogen receptor. Molecular docking of auraptene that is shown in green (**A**), endoxifen in purple (**B**), and 4-OH tamoxifen in light blue (**C**) on the ERα (PDB: 3ERT). ERα is shown in beige. Binding site residues and their types of interactions with auraptene (**D**), endoxifen (**E**), and 4-OH tamoxifen (**F**). (**G**) Comparison between MCF-7 and MCF-7^Var E^ under treatments with metabolites of tamoxifen and aurapteno for ER, Cateptsin D, eIF4A, eIF4G, and eIF4E. Densitometry analysis of ER (**H**) and eIF4G (**I**) in MCF-7 and MCF-7^Var E^ cells under the same conditions. Results are reported as mean ± SD (*n* = 3) and expressed as fold-change in regard to loading control; * *p* < 0.05, ** *p* < 0.002, *** *p* < 0.001 in regard to control. β-actin and Actin were used as loading controls.

Taking into account auraptene can modulate ER activity, impacting cellular processes such as proliferation, apoptosis, and, potentially, chemoresistance; we evaluated the possibility that the synergy observed between auraptene- and tamoxifen-derived metabolites could arise from auraptene ER binding. Therefore, a molecular docking simulation compared the binding positions of auraptene and tamoxifen metabolites to the natural ER ligand, estradiol (Figure 7A–F). In the first instance, estradiol and auraptene displayed similar affinity values or E-scores of −5.60 and −5.39, respectively. Notably, auraptene was found to bind the estradiol-denominated ligand-binding domain (LBD), suggesting potential competition for the estrogen receptors’ (ER) natural ligand-binding domain (Figure 7A–D). In this regard, auraptene shares several binding residues with estradiol, including Arg_394_, Glu_353_, Leu_346_, Leu_349_, Leu_384_, Leu_525_, Ile_424_, and Met_421_, resulting in a similar affinity value to estradiol.

Critically, the values of the E-scores for endoxifen and 4-OH Tam were −7.81 and −7.29, respectively (Figure 7A–F), and share some binding residues among them such as Glu_380_, Leu_536_, and Leu_525_ (Figure 7A–F). Trp_383_ and Leu_525_ are shared among auraptene and endoxifen binding sites, and Tyr_347_ and Leu_525_ are shared among 4-OH Tam and auraptene binding sites, suggesting that auraptene may exert similar activity as tamoxifen metabolites, which is a competitive inhibition of the ER, which remains to be confirmed with in vitro experiments.

We thus attempted to completely inhibit ER with the strategy of joint treatments of tamoxifen-derived metabolites and auraptene. However, these treatments are quite aggressive for the cellular models, as evidenced in Figure 5 and Figure 6; therefore, the incubation time was reduced from 12 h in the previous experiments to 6 h in order to obtain enough cellular lysates to perform Western blot assays (Figure 7G,H), as a result of significant cellular damage (Supplementary Figure S5). Under the joint treatments of active tamoxifen metabolites (8 μM) and auraptene (12.5 μM), we evaluated the ER, cathepsin D, and eIF4F protein expressions. The results indicate that simultaneous treatment of auraptene alongside tamoxifen metabolites exert a notable down-regulation effect on ERα in MCF-7 with a more pronounced effect in MCF-7^Var E^ variant (Figure 7G,H). In addition, a decrease in cathepsin D was also registered under the combined treatments. Then, the development of a potential therapeutic strategy is highlighted (Figure 7G,H).

Concomitant treatments also triggered the down-regulation of eIF4G, another eIF4F complex protein (Figure 7G,H), which may disrupt the assembly of the eIF4F complex. This effect could be further potentiated in MCF-7 cells, wherein eIF4E expression was also down-regulated under concomitant treatment of auraptene and 4-OH Tam (Figure 7H,I).

### 3.8. Auraptene Treatment Could Inhibit Chemoresistance to Tamoxifen Metabolites through Suppression of ABC Transport Expression

To broaden the characterization of the chemoresistance mechanism in MCF-7^Var E^ cells and the potential effect of auraptene and tamoxifen metabolites, we characterized the transcriptional modulation of the ABCB1 and ABCC1 transporter genes associated with the eIF4F translation complex. Under control conditions, MCF-7^Var E^ cells have expression levels almost twice as high as ABCB1 (Supplementary Figure S6A). In this regard, endoxifen severely alters the ABCB1 expression pattern, as MCF-7 cells display a 25-fold increase in expression, whereas MCF-7^Var E^ cells only show a slight expression decrease. The 4-OH Tam treatment promotes a slight increase in expression on both MCF-7 and MCF-7^Var E^ cells (Supplementary Figure S6A). In an interesting way, joint treatment of auraptene and endoxifen completely abates ABCB1 expression in both cellular variants (Supplementary Figure S6A). 

The ABCC1 transporter showed a similar expression pattern as ABCB1, as MCF-7^Var E^ cells also show almost two-fold expression levels of ABCC1. Both 4-OH Tam and auraptene single treatments trigger a 2.5-fold expression increase in MCF-7^Var E^ cells. However, mixed treatments of auraptene- and tamoxifen-derived metabolites abated ABCC1 expression on MCF-7 and MCF-7^Var E^ cells (Supplementary Figure S6B). The concomitant treatments demonstrate their highest efficacy once MCF-7^Var E^ acquires resistance; on the other hand, they up-regulate the expression when treating MCF-7 cells with endoxifen, and reduce the expression of ABCB1 and ABCC1.

### 3.9. Pharmacological Potential of Auraptene

As a perspective into an eventual use in clinical treatment, we performed an ADME analysis of auraptene from SwissADME (Supplementary Figure S7) and ADMETlab2.0, which provides an in silico analysis of a chemical compound’s properties and its potential as a drug. In this sense, auraptene has molecular weight of 298.16, a logP of 5.18, and a logS of −5.75, suggesting potential pharmaceutical properties based on its calculated solubility and permeability parameters [36]. It has moderately soluble characteristics according to ESOL and Ali models [35], suggesting its potential for adequate dissolution and absorption in biological systems. Its synthetic accessibility score (SA score) suggests simplicity for synthesis, and it adheres to the Golden Triangle rule, indicating a potentially favorable ADME profile [36].

In particular, auraptene displays a high gastrointestinal absorption, which indicates a favorable pharmacokinetic behavior. Moreover, auraptene shows no significant inhibition of key cytochrome P450 enzymes (CYP1A2, CYP2C19, CYP2C9, CYP2D6, and CYP3A4), suggesting its minimal potential for drug–drug interactions according to the ADME profile (Supplementary Figure S7). These findings suggest that auraptene cannot be ruled out as a compound for eventual pharmaceutical development.

## 4. Discussion

In the present work, tamoxifen-derived metabolite-resistant variant MCF-7^Var E^ was generated as a model to replicate the chemoresistant phenotype observed in patients with ER+ breast cancer, wherein tamoxifen is one of the main adjuvants used. In this regard, this model is a heterogeneous pool of cells, which is obtained through multiple processes of resistance generation. Although cellular models resistant to tamoxifen have been developed [40,41,42,43], resistant models to its primary plasmatic active metabolites, 4-OH Tam and endoxifen, have yet to be developed. In this context, a novel approach was taken in generating resistance by incorporating E2 into the 4–5 months resistance induction protocol (Figure 1A). This model closely mirrors physiological conditions that involve competition between tamoxifen metabolites and E2. As a result, we generated an endoxifen and 4-OH Tam-resistant MCF-7^Var E^ cellular model with higher EC_50_ values compared to the MCF-7 cell line.

We characterized the mechanisms associated with drug resistance and the main cellular features of the resistant cell line with evident phenotypic changes. In contrast to previous reports, our results indicate an ER expression increase in resistant cells. ER overexpression could have been driven by the metabolites used to induce resistance, given their ER agonist nature. In this regard, ER overexpression could act as a resistance mechanism, wherein the dual stimulation by tamoxifen metabolites and E2 triggers an increase in receptor expression. The MCF-7^Var E^ variant shows a distinct phenotype from the usual scenario, wherein drug resistance is associated with decreased ER expression [44,45]. 

The dose of tamoxifen metabolites was insufficient to decrease receptor activity. Furthermore, when using estradiol, which increases ER expression levels, a new phenotype was generated in which we registered that the growth rate of the resistant cell lines is lower compared to the parental cells, in addition to exhibiting slight morphological changes (Supplementary Figure S2).

On the other hand, we evaluated the effect of treatments with E2 on both MCF-7 and MCF-7^Var E^ cells, in which a protective E2 effect was expected. However, combined treatments with E2 plus tamoxifen metabolites resulted in a decrease in ER+ expression in resistant cells (Figure 2A). Due to the combined ER binding, diminished ER expression levels, and lower downstream signaling processes, significant cellular damage was observed through optical microscopy (Supplementary Figure S2) [46].

In the context of acquired resistance in MCF7^Var E^ cells and the ER expression increase, which is associated with higher protein translation levels [47], analyzing the eIF4F complex becomes relevant [9]. We observed an increase in the expression of translation initiation factors eIF4A and eIF4G in MCF7^Var E^ cells, which are crucial for the assembly of the translationally active ribosome. Given their crucial role in translation, these proteins have been identified as potential therapeutic targets. Natural compounds have shown therapeutic potential against the translation machinery, and compounds like rocaglamide have been proven to inhibit eIF4A [17]. It has also been observed that inhibiting eIF4A can reduce ER expression when combined with fulvestrant [16], showing an effect similar to our proposal involving auraptene and tamoxifen metabolites. Given its anticancer activity, we explored auraptene regulation of eIF4A activity.

In this regard, spectroscopy data from interaction assays support its binding capability, indicating a similar mechanism of activity to rocaglamide. However, the binding mechanism of auraptene to eIF4A remains to be elucidated. 

Auraptene has also been shown to have anticarcinogenic effects in different cancer cell lines [39]. It has been observed that auraptene exerts a series of anti-tumor effects, inducing apoptosis and the generation of reactive oxygen species (ROS) in colorectal cancer cells, as well as antiproliferative effects by inducing cell cycle arrest [48]. Thus, our data add an additional effect of auraptene and indicate a potential to be included in chemoresistant breast cancer treatment, but further experiments will be necessary.

In this regard, auraptene showed similar results on both the native and resistant cell lines, with an EC_50_ close to 50 μM for both cases. Due to this, combined treatments were performed with tamoxifen metabolites, wherein, surprisingly, we found a similar behavior in both cell lines, with effects observed even at the lowest concentration used in this assay, which was 25 μM for auraptene and 8 μM for the tamoxifen metabolites (Figure 5), suggesting a synergy between both compounds. Based on this, an assay was conducted with auraptene concentrations ranging from 0 to 35 μM to determine the minimum effective concentration. The combined treatments were found to be effective at a concentration of 12.5 μM (Figure 6). Similarly, a TNBC cell line was characterized to determine if the ER was required for the treatment to exert auraptene effects (Table 1). Critically, combined treatment was only effective for the ER+ and not for the TNBC, suggesting the specificity of the treatment of auraptene with tamoxifen metabolites (Table 1). With this evidence, it is worth noting that the proposed tamoxifen–auraptene treatment would have no effect on ER cell populations or populations which respond to treatment by down-regulating ER expression.

Notwithstanding, in the resistant cell MCF-7^Var E^, the therapeutic effect was more significant with the endoxifen and auraptene joint treatment (EC_50_ 6.47 μM), a phenomenon that could be caused by a synergism between the two compounds. The synergy between tamoxifen metabolites has already been reported by Chisholm, K and collaborators, where they observed synergy between epicatechins and metabolites derived from tamoxifen in the MDA-MB-231 cell line [49] and attributed the effect to the sensitization of the apoptosis process [50].

A down-regulation effect on ERα was observed in MCF-7 and MCF-7^Var E^ variant cells under the joint treatments of auraptene and tamoxifen metabolites, suggesting a potential for complete ER inhibition (Figure 7G). Interestingly, the concomitant treatments also led to a decrease in cathepsin D and eIF4G expression, critical proteins involved in cellular processes. This down-regulation of eIF4G could disrupt the assembly of the eIF4F complex, further impacting cellular functions. These findings represent a first step in proving that the ER activity and eIF4F components could be modulated by auraptene and be exploited as a therapeutic strategy for breast cancer treatment. Further experiments are needed to exactly determine the inhibition mechanism of this compound on eIF4A.

In a complementary way, we broadly elucidate the drug resistance mechanism in MCF-7^Var E^ cells and its potential regulation by auraptene and tamoxifen metabolites. We focused on characterizing the transcriptional modulation of transporter genes associated with the eIF4F translation complex [51] and ABCB1 and ABCC1 transporters. The combined treatment of auraptene and 4-OH tamoxifen completely diminish ABCB1 expression in MCF-7^Var E^ while maintaining basal levels in MCF-7 cells (Supplementary Figure S5). Similarly, joint treatments profoundly impacted ABCC1 expression, leading to a significant decrease in expression levels in both cell lines. This phenomenon suggests that auraptene, in combination with tamoxifen metabolites, may exert regulatory effects on ABCB1 and ABCC1 transporter expression.

## 5. Conclusions

Our study presents a novel approach to design treatments for chemoresistance in an ER+ cellular model. We discovered a previously unreported mechanism involving the participation of estradiol (E2) in the development of chemoresistance under an equimolar scheme with active tamoxifen-derived metabolites. This unique condition suggests an increment of estrogen receptor (ER) activity and the participation of the eIF4F complex. Also, auraptene may be a potential candidate for modulating eIF4A activity in addition to ER, hampering key factors of cell translation and proliferation. It is worth mentioning that this phenomenon presents specificity for the ER+ phenotype, considering the results in the TNBC model. Interestingly, combined treatments of auraptene and tamoxifen metabolites resulted in a significant down-regulation of ER expression, suggesting a potential therapeutic strategy to overcome chemoresistance.

## 6. Patents

The experimental analysis is protected under Mexico law MX/E/2024/037190.

## Figures and Tables

**Table 1:** EC_50_ values for ER+ and TNBC under auraptene and tamoxifen metabolite treatments. Values for the combined treatments in different cell variants.

Cell Variant	Characteristic	Auraptene (µM)	Auraptene + 4-OH Tam (µM)	Auraptene + Endoxifen (µM)
MCF-7	ER+	47.49	12.69	13.34
MCF-7^Var E^	Drug resistance	52.40	8.53	6.47
MDA	TNBC	76.75	80.65	52.68

## Data Availability

Data are contained within the article.

## Supplementary Materials

The following supporting information can be downloaded at: https://www.mdpi.com/article/10.3390/pharmaceutics16091179/s1, Figure S1. Resistance development in MCF-7 cells. Figure S2. Effect of estradiol in MCF-7 cells. Figure S3. Docking of estrogen receptor. Figure S4. Effect of auraptene on the eIF4A regulation. Figure S5. Effect of auraptene and joint treatments in MCF-7 cells and MCF-7^Var E^. Figure S6. Modulation of the gene expression of ABCC1 and ABCB1. Figure S7. SwissADME results on auraptene properties.
