# Supplementary material for: Auraptene Boosts the Efficacy of the Tamoxifen Metabolites Endoxifen and 4-OH-Tamoxifen in a Chemoresistant ER+ Breast Cancer Model
Source: Pharmaceutics. 2024 Sep 6;16(9):1179. doi: 10.3390/pharmaceutics16091179 (PMC11435248; doi:10.3390/pharmaceutics16091179)
Supplement: Supplementary file 1 [file pharmaceutics-16-01179-s001.zip › pharmaceutics-3118750-supplementary.pdf]

Supplementary Materials

Auraptene Boosts the Efficacy of the Tamoxifen Metabolites Endoxifen and 4-OH-Tamoxifen in a Chemoresistant ER+ Breast Cancer Model

Angel Pulido-Capiz [1,2], Brenda Chimal-Vega [1,2], Luis Pablo Avila-Barrientos [3], Alondra Campos-Valenzuela [1,2], Raúl Díaz-Molina [1,2], Raquel Muñiz-Salazar [4], Octavio Galindo-Hernández [1,2] and Victor García-González [1,2,*]

[1] Departamento de Bioquímica, Facultad de Medicina Mexicali, Universidad Autónoma de Baja California, Mexicali 21000, Mexico

[2] Laboratorio Multidisciplinario de Estudios Metabólicos y Cáncer, Universidad Autónoma de Baja California, Mexicali 21000, Mexico

[3] Max-Planck-Institute of Molecular Plant Physiology, Am Mühlenberg 1, 14476 Potsdam, Germany

[4] Escuela de Ciencias de la Salud, Universidad Autónoma de Baja California, Campus Ensenada, Ensenada 22890, Mexico

* Correspondence: vgarcia62@uabc.edu.mx

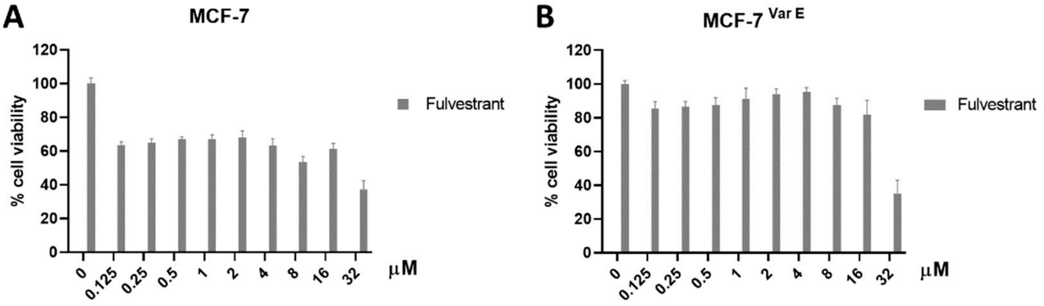

C

IC_{50}	Fulvestrant (μM)
MCF-7	19.81 ± 0.49
MCF-7$^{Var E}$	29.62 ± 0.49

Supplementary Figure S1. Resistance development in MCF-7 cells. (**A**) Viability percentages of MCF-7, (**B**) and MCF-7$^{Var E}$ cells treated under increasing treatment concentrations of Fulvestrant. (0–32 μM) for 24 h. (**C**) Comparison of the IC50 values among MCF-7 and MCF-7var E cells under Fulvestrant treatments.

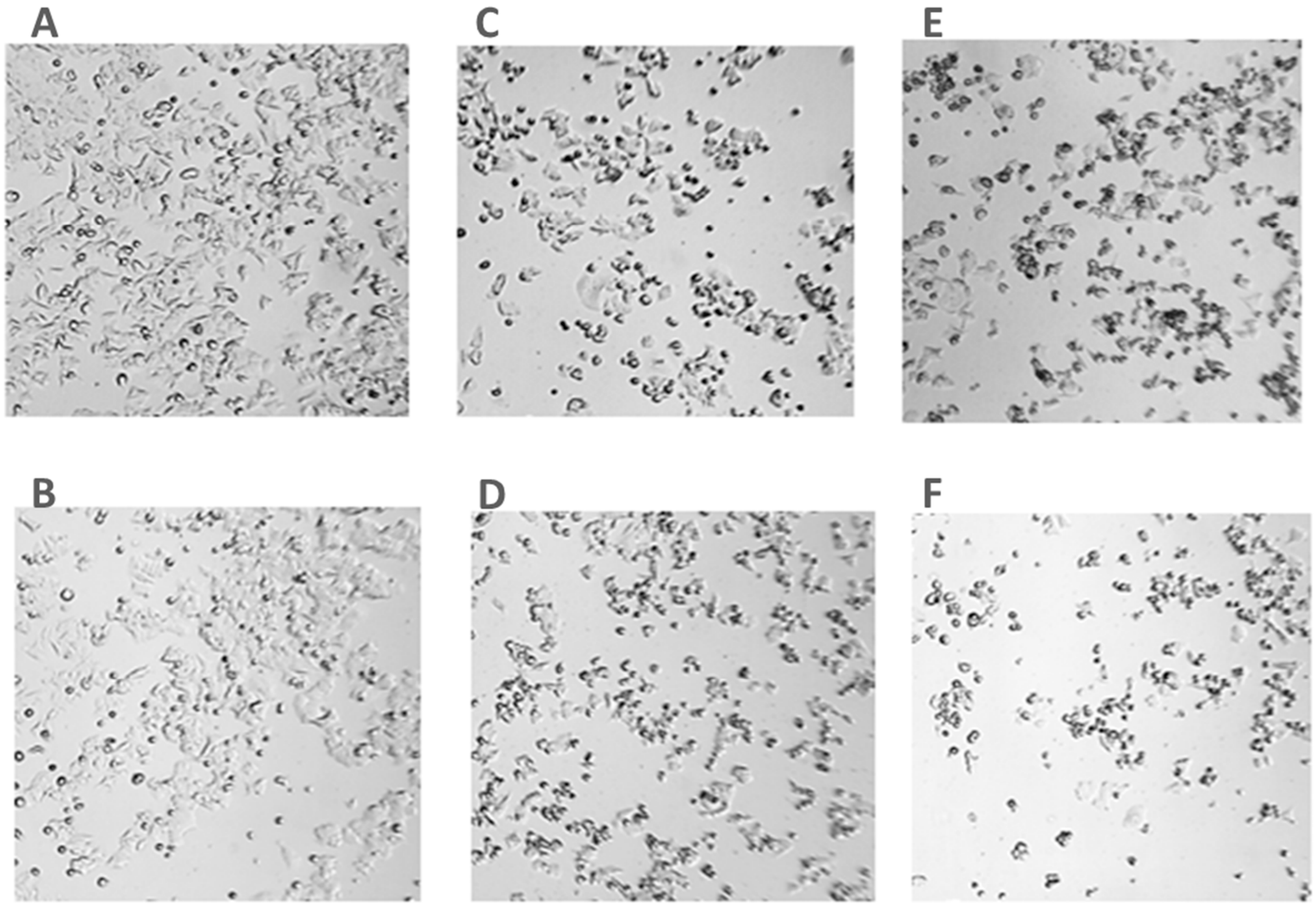

Supplementary Figure S2. Effect of estradiol in MCF-7 cells. Comparative between treatments with E2 using optic microscopy at 10× showing an increase in damage with joint treatments with tamoxifen metabolites in 12 h. Images corresponding to Control (**A**), Estradiol 2 µM (**B**), Endoxifen 8 µM (**C**), Endoxifen 8 µM + E2 2 µM (**D**), 4-OH Tam 8 µM (**E**)**,** 4-OH Tam 8 µM+ E2 2 µM (**F**).

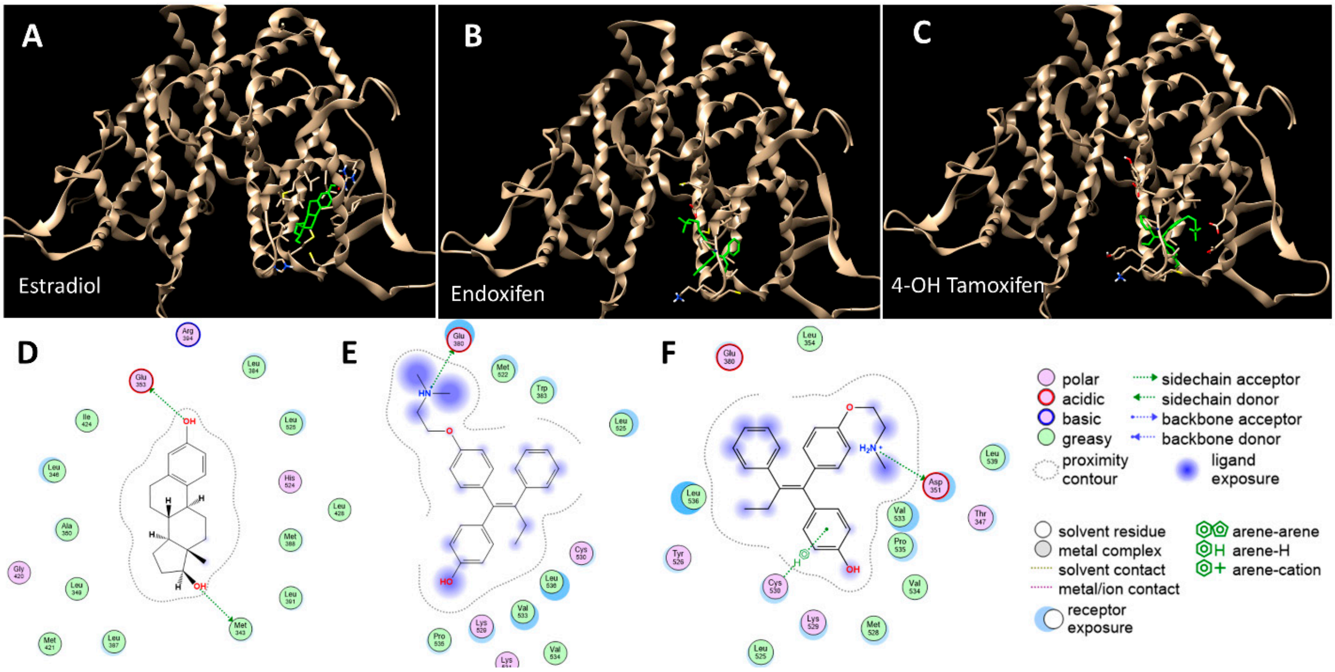

Supplementary Figure S3. Docking of Estrogen receptor. Estrogen receptor crystal in color beige (PDB: 3ERT). (**A**) Estrogen receptor crystal and Estradiol in color green. (**B**) Estrogen receptor crystal plus endoxifen (green) site of interaction. (**C**) Estrogen receptor crystal plus 4-OH tamoxifen (green) site of interaction. (**D**) The ligand interaction of estradiol shows the ER residues and the type of interaction. (**E**) Ligand interaction of endoxifen shows the ER residues and type of interaction. (**F**) Ligand interaction of 4-OH tamoxifen shows the ER residues and type of interaction.

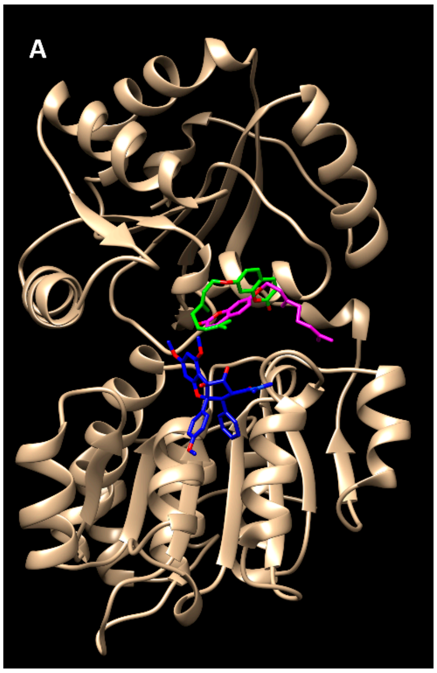

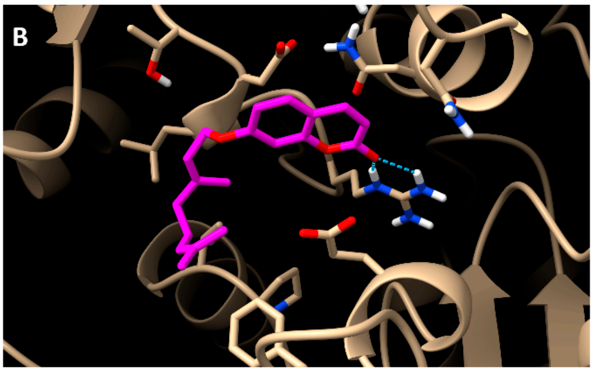

C

Compound	MOE Score	Vina Score
Rocaglamide	-10.2	-9.6
Auraptene	-9.7	-6.3

Supplementary Figure S4. Effect of Auraptene on the eIF4A regulation. (**A**) Binding poses of Auraptene found by MOE and Vina are shown in magenta and green, respectively. Also, Rocaglamide from the 5zc9 structure (PDB) is shown in blue. (**B**) Binding pose found by Vina is shown with surrounding residues shown. The potential hydrogen bond with Arg334 is shown in light blue. (**C**) Binding scores obtained in MOE and Vina.

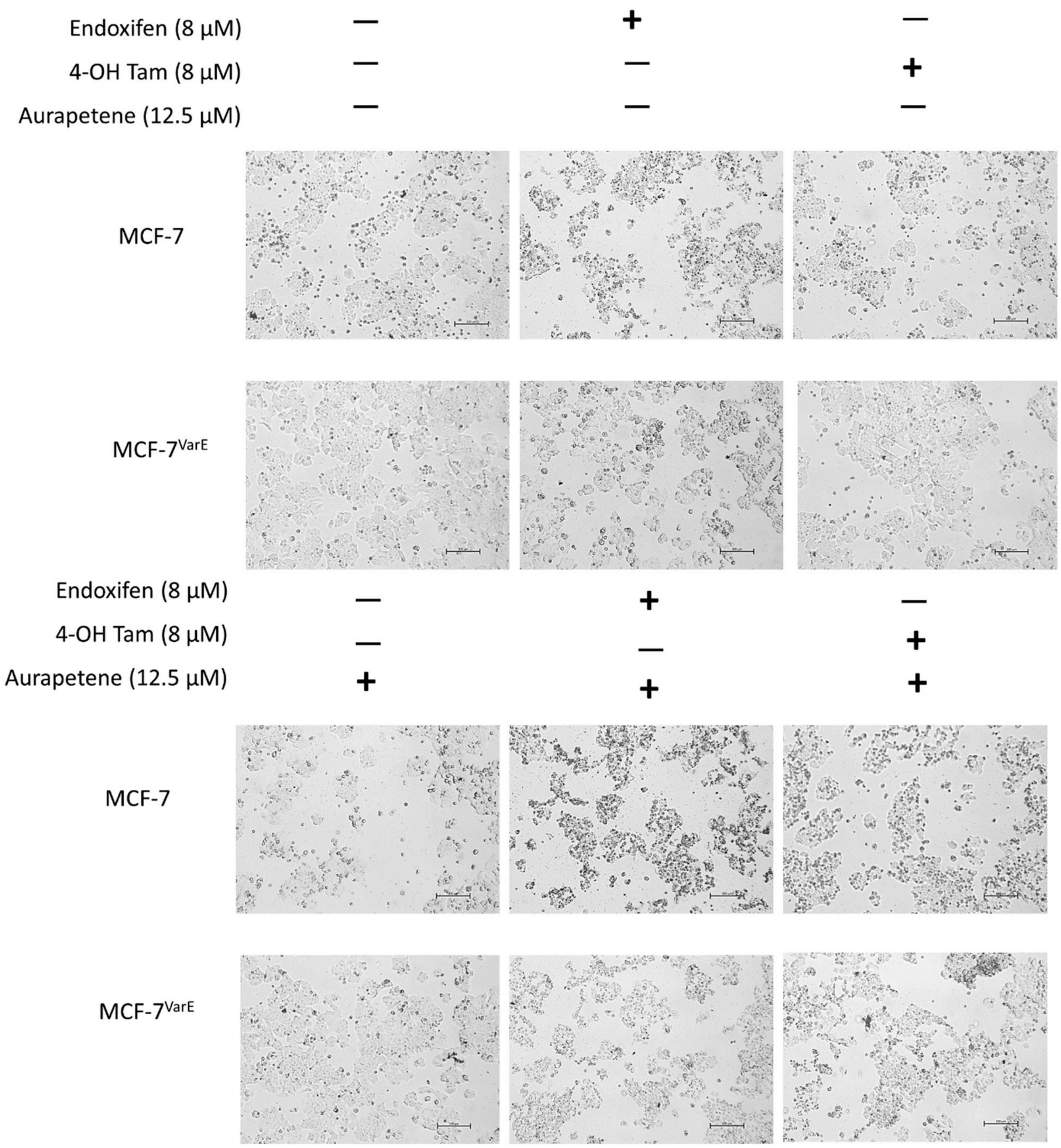

Supplementary Figure S5. Effect of Auraptene and joint treatments in MCF-7 cells and MCF-7$^{Var\ E}$. Comparative between treatments with Auraptene plus tamoxifen metabolites, using optic microscopy showing the damage with joint treatments with tamoxifen metabolites in a time curse of 12 h at 10× increment.

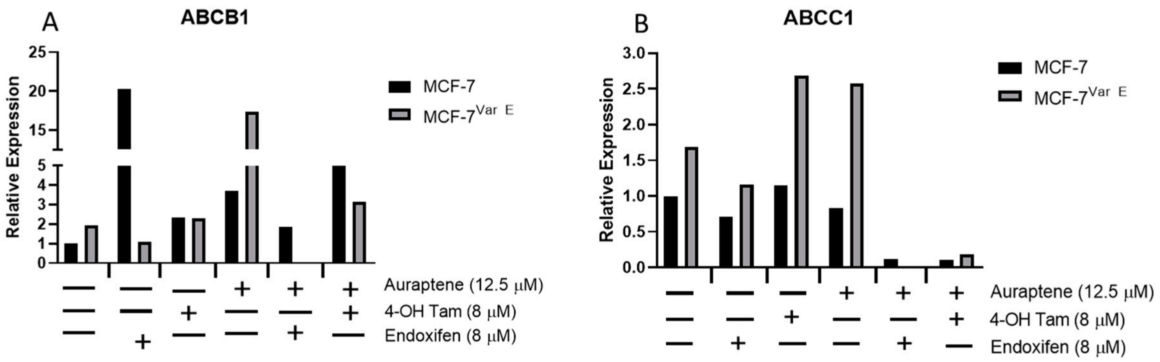

Supplementary Figure S6. Modulation of the gene expression of ABCC1 and ABCB1. Comparative between treatments with Auraptene (12.5 µM) plus tamoxifen metabolites (8 µM) in MCF-7 and MCF-7$^{Var\ E}$ cells. (**A**) Relative gene expression of ABCB1, in black MCF-7, in grey MCF-7$^{Var\ E}$. (**B**) Relative gene expression of ABCC1. Relative expression of ABCC1 normalized with GAPDH, in black MCF-7, in grey MCF-7$^{Var\ E}$.

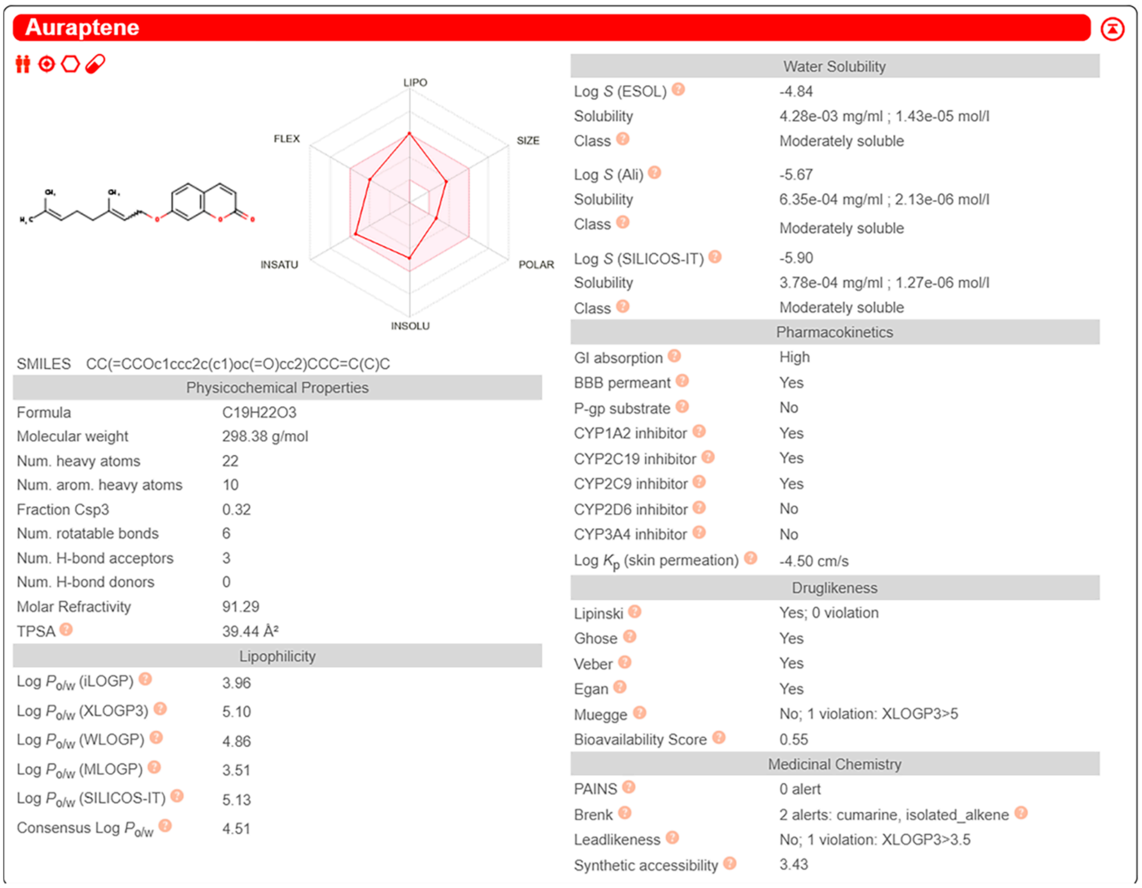

Supplementary Figure S7. SwissADME results on Auraptene properties. The result can be calculated following the instructions above using the data source SwissADME (http://www.swissadme.ch/index.php/; Swiss Institute of Bioinformatics; accessed on 18 March 2024).
